# Crystal structure of γ-ethyl-l-glutamate *N*-carb­oxy anhydride

**DOI:** 10.1107/S2056989014027170

**Published:** 2015-01-01

**Authors:** Hitoshi Kanazawa, Aya Inada

**Affiliations:** a Faculty of Symbiotic Systems Science, Fukushima University, 1 Kanayagawa, Fukushima, 960-1296, Japan

**Keywords:** crystal structure, solid-state polymerization, amino acid *N*-carb­oxy anhydrides, hydrogen bonding

## Abstract

In the title compound (alternative name *N*-carboxy-l-glutamic anhydride γ-ethyl ester), C_8_H_11_NO_5_, the oxazolidine ring is essentially planar, with a maximum deviation of 0.019 (2) Å. In the crystal, mol­ecules are linked by N—H⋯O hydrogen bonds between the imino group and the carbonyl O atom in the ethyl ester group, forming a tape structure along the *c-*axis direction. The oxazolidine rings of adjacent tapes are arranged into a layer parallel to the *ac* plane. This arrangement is favourable for the polymerization of the title compound in the solid state.

## Abstract 2

In the crystal of the title compound, mol­ecules are linked by N—H⋯O hydrogen bonds between the imino group and the carbonyl O atom in the ethyl ester group, forming a tape structure along the *c*-axis direction. The oxazolidine rings of adjacent tapes are arranged into a layer parallel to the *ac* plane, which is a favourable arrangement for the polymerization of the title compound in the solid state.

## Chemical context   

*N*-Carb­oxy anhydrides (NCAs) of amino acids are extensively used as monomers in the preparation of high mol­ecular weight polypeptides (Kricheldorf, 2006 ▸). Amino acid NCAs are easily soluble but the resulting polypeptides are not soluble in general organic solvents. Only a few amino acid ester NCAs such as γ-benzyl-l-glutamate NCA and β-benzyl-l-aspartate NCA can be polymerized in solutions, because the resulting polypeptides are soluble in them. Thus, the polymerization of these amino acid ester NCAs has been investigated by many researchers. We found that every amino acid NCA crystal is polymerized in the solid state in hexane by the initiation of amines, and have studied the solid-state polymerization of amino acid NCAs with reference to the crystal structures (Kanazawa, 1992 ▸; Kanazawa & Magoshi, 2003 ▸; Kanazawa *et al.*, 2006 ▸).
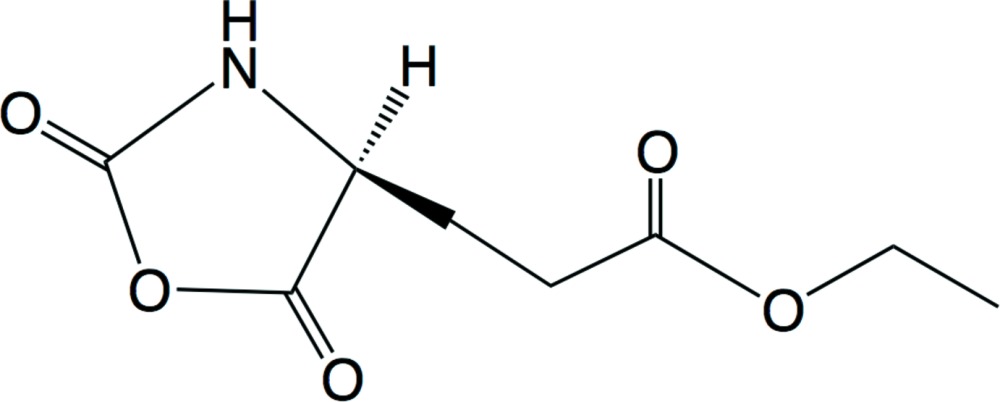


The title compound, γ-ethyl-l-glutamate NCA (ELG NCA) is polymerized both in dioxane solution and in the solid state in hexane, using butyl­amine as initiator. However, ELG NCA is very reactive in the solid state in hexane using the same initiator. Therefore, it is important to determine the crystal structure in order to consider the difference in the reactivity of ELG NCA in solution and in the solid state.

## Structural commentary   

**Figure 1:**
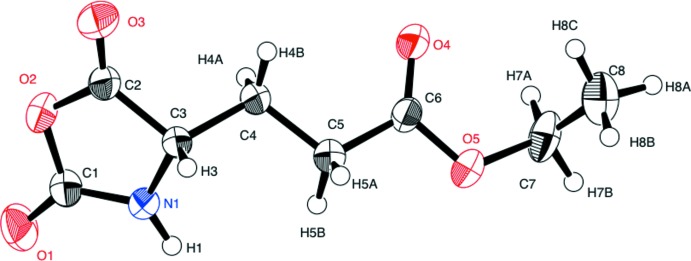
The mol­ecular structure of the title compound showing atom labels and 50% probability displacement ellipsoids for non-H atoms.

The mol­ecular structure of the title compound is shown in Fig. 1 ▸. The oxazolidine ring is essentially planar, with a maximum deviation of 0.019 (2) Å for atom C1. The side chain has an extended conformation with the torsion angles C3—C4—C5—C6 and C4—C5—C6—O5 being 177.65 (13) and −172.05 (13)°, respectively.

## Supra­molecular features   

**Figure 2:**
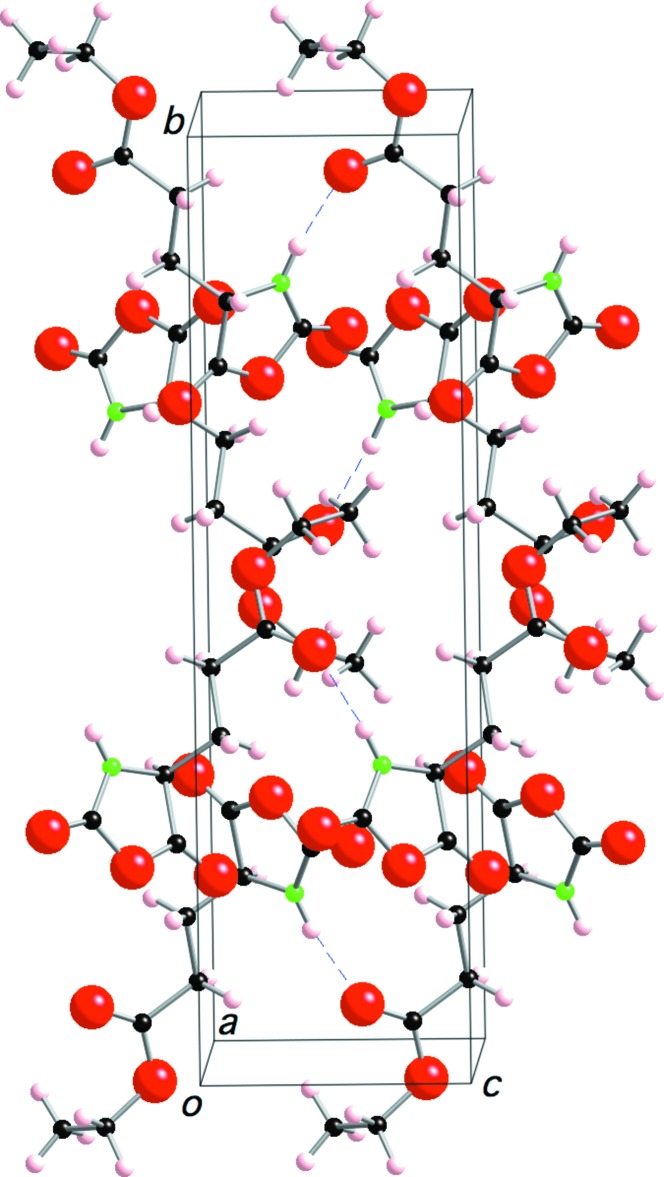
A crystal packing diagram of the title compound, viewed approximately along the *a* axis, showing the hydrogen bonds as dashed lines (see Table 1 ▸ for details).

In the crystal, ELG NCA mol­ecules are linked by N1—H1⋯O4 hydrogen bonds along the *c* axis (Table 1 ▸ and Fig. 2 ▸). The five-membered oxazolidine rings are packed in a layer and the –CH_2_CH_2_COOCH_2_CH_3_ groups are packed in another layer; these two different layers are stacked alternately. This sandwich structure is one of the important requirements for high reactivity in the solid state, because the five-membered rings can react with each other in the layer.

## Database survey   

**Figure 3:**
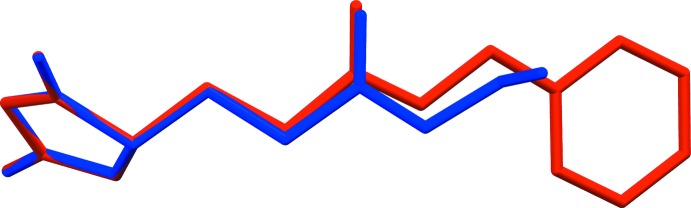
A view of the structural overlay of the title compound (blue) and ANCBGL (red; Kanazawa *et al.*, 1978 ▸).

A search of the Cambridge Structural Database (Version 5.35, May 2014; Groom & Allen, 2014 ▸) revealed the presence of 20 hits for 4-methyl­oxazolidine-2,5-dione derivatives. A number of these compounds involve amino acid sides chains (amino acid NCAs). These include two polymorphs of a compound involving l-aspartate, namely *N*-carb­oxy-β-benzyl-l-aspartate anhydride (SOHRIQ: Kanazawa, 1998 ▸; no coordinates were deposited) and (SOHRIQ01: Kanazawa & Magoshi, 2003 ▸). Two other compounds involving l-glutamate have also been reported. They are very similar to the title compound and are polymorphs of *N*-carb­oxy-γ-benzyl-l-glutamate anhydride (ANCBGL; Kanazawa *et al.*, 1978 ▸) and (WIPDUV; Kanazawa *et al.*, 2006 ▸). For the latter, unfortunately no coordinates have been deposited. The structural overlay of the title compound and ANCBGL indicates that the *N*-carb­oxy-l-glutamate anhydride moieties have very similar conformations (Fig. 3 ▸).

## Synthesis and crystallization   

The synthesis of γ-ethyl-l-glutamate (ELG) was carried out by the reaction of l-glutamic acid with ethanol in a manner similar to that of γ-benzyl-l-glutamate (BLG) (Kanazawa, 1992 ▸). The title compound was obtained by the reaction of γ-ethyl-l-glutamate with tri­chloro­methyl chloro­formate or triphosgene in tetra­hydro­furan, as reported previously for β-benzyl-l-aspartate (BLA) NCA (Kanazawa & Magoshi, 2003 ▸). The reaction product was recrystallized in a mixture of ethyl acetate and hexane (1:50 *v*/*v*), avoiding moisture contamination.

## Refinement details   

Crystal data, data collection and structure refinement details are summarized in Table 2 ▸. The N-bound H atom was found in a difference Fourier map and its position was refined with *U*_iso_(H) = 1.5*U*_eq_(N). C-bound H atoms were positioned geometrically (C—H = 0.96–0.98 Å) and treated as riding with *U*_iso_(H) = 1.2*U*_eq_(C).

## Supplementary Material

Crystal structure: contains datablock(s) I, New_Global_Publ_Block. DOI: 10.1107/S2056989014027170/is5383sup1.cif

Structure factors: contains datablock(s) I. DOI: 10.1107/S2056989014027170/is5383Isup2.hkl

Supporting information file. DOI: 10.1107/S2056989014027170/is5383Isup3.cml

CCDC reference: 1038820

Additional supporting information:  crystallographic information; 3D view; checkCIF report

## Figures and Tables

**Table 1:** Hydrogen-bond geometry (, )

*D*H*A*	*D*H	H*A*	*D* *A*	*D*H*A*
N1H1O4^i^	0.76(2)	2.13(2)	2.8766(17)	170(2)

Symmetry code: (i) 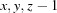.

**Table 2:** Experimental details

Crystal data	
Chemical formula	C_8_H_11_NO_5_
*M*_r_	201.18
Crystal system, space group	Orthorhombic, *P*2_1_2_1_2
Temperature (K)	293
*a*, *b*, *c* ()	7.9337(19), 20.581(5), 5.8405(14)
*V* (^3^)	953.7(4)
*Z*	4
Radiation type	Mo *K*
(mm^1^)	0.12
Crystal size (mm)	0.66 0.39 0.14

Data collection	
Diffractometer	Rigaku XtaLAB mini
Absorption correction	Multi-scan (*REQAB*; Rigaku, 1998 ▸)
*T*_min_, *T*_max_	0.926, 0.984
No. of measured, independent and observed [*I* > 2(*I*)] reflections	9982, 2190, 2042
*R*_int_	0.024
(sin /)_max_ (^1^)	0.649

Refinement	
*R*[*F* ^2^ > 2(*F* ^2^)], *wR*(*F* ^2^), *S*	0.034, 0.081, 1.03
No. of reflections	2190
No. of parameters	131
H-atom treatment	H atoms treated by a mixture of independent and constrained refinement
_max_, _min_ (e ^3^)	0.16, 0.16

Computer programs: *CrystalClear* (Rigaku, 2009 ▸), *SIR2004* (Burla *et al.*, 2005 ▸), *SHELXL97* (Sheldrick, 2008 ▸), *CrystalStructure* (Rigaku, 2010 ▸) and *Mercury* (Macrae *et al.*, 2008 ▸).

## supplementary crystallographic information

### Crystal data

**Table:**

C_8_H_11_NO_5_	*F*(000) = 424
*M**_r_* = 201.18	*D*_x_ = 1.401 Mg m^−^^3^
Orthorhombic, *P*2_1_2_1_2	Mo *K*α radiation, λ = 0.71069 Å
Hall symbol: P 2 2ab	θ = 3.2–27.5°
*a* = 7.9337 (19) Å	µ = 0.12 mm^−^^1^
*b* = 20.581 (5) Å	*T* = 293 K
*c* = 5.8405 (14) Å	Prism, colorless
*V* = 953.7 (4) Å^3^	0.66 × 0.39 × 0.14 mm
*Z* = 4	

### Data collection

**Table:**

Rigaku XtaLAB mini diffractometer	2190 independent reflections
Radiation source: fine-focus sealed tube	2042 reflections with *I* > 2σ(*I*)
Graphite monochromator	*R*_int_ = 0.024
Detector resolution: 6.827 pixels mm^-1^	θ_max_ = 27.5°, θ_min_ = 3.2°
ω scans	*h* = −10→10
Absorption correction: multi-scan (*REQAB*; Rigaku, 1998)	*k* = −26→26
*T*_min_ = 0.926, *T*_max_ = 0.984	*l* = −7→7
9982 measured reflections	

### Refinement

**Table:**

Refinement on *F*^2^	Primary atom site location: structure-invariant direct methods
Least-squares matrix: full	Secondary atom site location: difference Fourier map
*R*[*F*^2^ > 2σ(*F*^2^)] = 0.034	Hydrogen site location: inferred from neighbouring sites
*wR*(*F*^2^) = 0.081	H atoms treated by a mixture of independent and constrained refinement
*S* = 1.03	*w* = 1/[σ^2^(*F*_o_^2^) + (0.0385*P*)^2^ + 0.167*P*] where *P* = (*F*_o_^2^ + 2*F*_c_^2^)/3
2190 reflections	(Δ/σ)_max_ = 0.010
131 parameters	Δρ_max_ = 0.16 e Å^−^^3^
0 restraints	Δρ_min_ = −0.16 e Å^−^^3^

### Special details

**Table:**

Geometry. All e.s.d.'s (except the e.s.d. in the dihedral angle between two l.s. planes) are estimated using the full covariance matrix. The cell e.s.d.'s are taken into account individually in the estimation of e.s.d.'s in distances, angles and torsion angles; correlations between e.s.d.'s in cell parameters are only used when they are defined by crystal symmetry. An approximate (isotropic) treatment of cell e.s.d.'s is used for estimating e.s.d.'s involving l.s. planes.
Refinement. Refinement of *F*^2^ against ALL reflections. The weighted *R*-factor *wR* and goodness of fit *S* are based on *F*^2^, conventional *R*-factors *R* are based on *F*, with *F* set to zero for negative *F*^2^. The threshold expression of *F*^2^ > σ(*F*^2^) is used only for calculating *R*-factors(gt) *etc*. and is not relevant to the choice of reflections for refinement. *R*-factors based on *F*^2^ are statistically about twice as large as those based on *F*, and *R*- factors based on ALL data will be even larger.

### Fractional atomic coordinates and isotropic or equivalent isotropic displacement parameters (Å^2^)

**Table:**

	*x*	*y*	*z*	*U*_iso_*/*U*_eq_	
O1	−0.02731 (16)	0.77067 (6)	−0.0480 (2)	0.0538 (4)	
O2	0.10460 (14)	0.72562 (4)	0.25649 (18)	0.0385 (3)	
O3	0.27691 (18)	0.70878 (5)	0.5536 (2)	0.0548 (4)	
O4	0.19733 (14)	0.94889 (5)	0.93375 (19)	0.0417 (3)	
O5	0.26952 (18)	1.03085 (5)	0.7069 (2)	0.0539 (4)	
N1	0.15491 (17)	0.82887 (6)	0.1795 (2)	0.0344 (3)	
C1	0.06782 (19)	0.77729 (7)	0.1092 (3)	0.0355 (3)	
C2	0.22322 (19)	0.74470 (6)	0.4126 (3)	0.0340 (3)	
C3	0.26475 (17)	0.81536 (6)	0.3704 (2)	0.0283 (3)	
C4	0.23203 (19)	0.85660 (6)	0.5828 (3)	0.0318 (3)	
C5	0.2829 (3)	0.92684 (7)	0.5461 (3)	0.0425 (4)	
C6	0.24451 (18)	0.96852 (6)	0.7513 (3)	0.0336 (3)	
C7	0.2257 (3)	1.07707 (7)	0.8886 (4)	0.0504 (5)	
C8	0.3640 (3)	1.08564 (9)	1.0556 (4)	0.0575 (5)	
H1	0.156 (3)	0.8596 (10)	0.108 (4)	0.0517*	
H3	0.3827	0.8197	0.3231	0.0339*	
H4A	0.1132	0.8547	0.6210	0.0381*	
H4B	0.2950	0.8389	0.7108	0.0381*	
H5A	0.4027	0.9288	0.5139	0.0509*	
H5B	0.2234	0.9439	0.4140	0.0509*	
H7A	0.1261	1.0617	0.9682	0.0604*	
H7B	0.1990	1.1188	0.8204	0.0604*	
H8A	0.4623	1.1017	0.9782	0.0689*	
H8B	0.3895	1.0446	1.1259	0.0689*	
H8C	0.3301	1.1161	1.1711	0.0689*	

### Atomic displacement parameters (Å^2^)

**Table:**

	*U*^11^	*U*^22^	*U*^33^	*U*^12^	*U*^13^	*U*^23^
O1	0.0582 (7)	0.0549 (7)	0.0482 (7)	−0.0087 (6)	−0.0192 (6)	−0.0092 (6)
O2	0.0425 (6)	0.0261 (4)	0.0470 (6)	−0.0067 (4)	−0.0017 (5)	−0.0028 (5)
O3	0.0817 (9)	0.0355 (5)	0.0473 (6)	0.0005 (6)	−0.0118 (7)	0.0098 (5)
O4	0.0598 (7)	0.0274 (4)	0.0380 (6)	−0.0052 (5)	0.0094 (6)	−0.0030 (4)
O5	0.0899 (9)	0.0257 (5)	0.0461 (6)	−0.0118 (6)	0.0003 (7)	0.0003 (5)
N1	0.0486 (7)	0.0267 (5)	0.0280 (5)	−0.0060 (5)	−0.0048 (5)	0.0024 (5)
C1	0.0387 (7)	0.0328 (7)	0.0349 (7)	−0.0035 (6)	−0.0014 (6)	−0.0044 (6)
C2	0.0431 (7)	0.0275 (6)	0.0313 (7)	−0.0004 (6)	0.0049 (7)	−0.0027 (6)
C3	0.0323 (6)	0.0269 (6)	0.0256 (6)	−0.0043 (5)	0.0016 (5)	−0.0021 (5)
C4	0.0411 (7)	0.0278 (6)	0.0264 (6)	−0.0037 (5)	0.0040 (6)	−0.0032 (5)
C5	0.0633 (10)	0.0333 (7)	0.0308 (7)	−0.0130 (7)	0.0052 (8)	−0.0035 (6)
C6	0.0402 (7)	0.0245 (6)	0.0361 (7)	−0.0054 (5)	−0.0046 (6)	0.0002 (5)
C7	0.0536 (9)	0.0243 (6)	0.0732 (11)	0.0023 (7)	−0.0051 (9)	−0.0090 (7)
C8	0.0606 (10)	0.0450 (9)	0.0668 (12)	0.0006 (8)	−0.0007 (10)	−0.0164 (9)

### Geometric parameters (Å, º)

**Table:**

O1—C1	1.1964 (18)	C4—C5	1.5159 (18)
O2—C2	1.3677 (18)	C4—H4A	0.9700
O2—C1	1.3985 (18)	C4—H4B	0.9700
O3—C2	1.1861 (17)	C5—C6	1.505 (2)
O4—C6	1.1997 (18)	C5—H5A	0.9700
O5—C6	1.3237 (15)	C5—H5B	0.9700
O5—C7	1.467 (2)	C7—C8	1.478 (3)
N1—C1	1.3314 (18)	C7—H7A	0.9700
N1—C3	1.4423 (18)	C7—H7B	0.9700
N1—H1	0.76 (2)	C8—H8A	0.9600
C2—C3	1.5113 (17)	C8—H8B	0.9600
C3—C4	1.5251 (17)	C8—H8C	0.9600
C3—H3	0.9800		
			
C2—O2—C1	109.58 (10)	H4A—C4—H4B	107.9
C6—O5—C7	116.82 (14)	C6—C5—C4	112.17 (12)
C1—N1—C3	113.46 (12)	C6—C5—H5A	109.2
C1—N1—H1	119.9 (15)	C4—C5—H5A	109.2
C3—N1—H1	125.7 (15)	C6—C5—H5B	109.2
O1—C1—N1	130.91 (15)	C4—C5—H5B	109.2
O1—C1—O2	121.12 (13)	H5A—C5—H5B	107.9
N1—C1—O2	107.97 (12)	O4—C6—O5	123.17 (14)
O3—C2—O2	122.09 (13)	O4—C6—C5	125.34 (12)
O3—C2—C3	129.38 (14)	O5—C6—C5	111.49 (13)
O2—C2—C3	108.53 (11)	O5—C7—C8	112.25 (14)
N1—C3—C2	100.35 (11)	O5—C7—H7A	109.2
N1—C3—C4	114.75 (11)	C8—C7—H7A	109.2
C2—C3—C4	111.48 (11)	O5—C7—H7B	109.2
N1—C3—H3	110.0	C8—C7—H7B	109.2
C2—C3—H3	110.0	H7A—C7—H7B	107.9
C4—C3—H3	110.0	C7—C8—H8A	109.5
C5—C4—C3	111.76 (11)	C7—C8—H8B	109.5
C5—C4—H4A	109.3	H8A—C8—H8B	109.5
C3—C4—H4A	109.3	C7—C8—H8C	109.5
C5—C4—H4B	109.3	H8A—C8—H8C	109.5
C3—C4—H4B	109.3	H8B—C8—H8C	109.5
			
C3—N1—C1—O1	−176.31 (16)	O3—C2—C3—C4	−57.2 (2)
C3—N1—C1—O2	3.49 (17)	O2—C2—C3—C4	122.05 (12)
C2—O2—C1—O1	176.53 (14)	N1—C3—C4—C5	−70.08 (16)
C2—O2—C1—N1	−3.30 (16)	C2—C3—C4—C5	176.72 (13)
C1—O2—C2—O3	−178.76 (14)	C3—C4—C5—C6	177.65 (13)
C1—O2—C2—C3	1.89 (15)	C7—O5—C6—O4	−4.1 (2)
C1—N1—C3—C2	−2.23 (15)	C7—O5—C6—C5	176.04 (14)
C1—N1—C3—C4	−121.83 (14)	C4—C5—C6—O4	8.0 (2)
O3—C2—C3—N1	−179.18 (16)	C4—C5—C6—O5	−172.05 (13)
O2—C2—C3—N1	0.11 (14)	C6—O5—C7—C8	84.9 (2)

### Hydrogen-bond geometry (Å, º)

**Table:**

*D*—H···*A*	*D*—H	H···*A*	*D*···*A*	*D*—H···*A*
N1—H1···O4^i^	0.76 (2)	2.13 (2)	2.8766 (17)	170 (2)

Symmetry code: (i) *x*, *y*, *z*−1.
